# Peripheral Blood Mononuclear Cells Cytokine Profile in a Patient with Toxic Epidermal Necrolysis Triggered by Lamotrigine and COVID-19: A Case Study

**DOI:** 10.3390/ijms26031374

**Published:** 2025-02-06

**Authors:** Margarita L. Martinez-Fierro, Idalia Garza-Veloz, Sidere Monserrath Zorrilla-Alfaro, Andrés Eduardo Campuzano-Garcia, Monica Rodriguez-Borroel

**Affiliations:** 1 Molecular Medicine Laboratory, Unidad Academica de Medicina Humana y Ciencias de la Salud, Universidad Autonoma de Zacatecas, Zacatecas C.P. 98160, Mexico; idaliagv@uaz.edu.mx (I.G.-V.); siderezorrilla@gmail.com (S.M.Z.-A.); 2 Secretaria de Salud de Zacatecas, Hospital General Zacatecas “Luz Gónzalez Cosío”, Zacatecas C.P. 98160, Mexico; campuzano3@hotmail.com

**Keywords:** Stevens–Johnson, toxic epidermal necrolysis, COVID-19, immune response, rare disease

## Abstract

Stevens–Johnson Syndrome (SJS)/toxic epidermal necrolysis (TEN) is a severe mucocutaneous reaction often induced by medications. The co-occurrence of SJS/TEN and COVID-19 presents a unique challenge due to overlapping inflammatory pathways. This case study examined the cytokine profile of a patient with both TEN (triggered by lamotrigine) and COVID-19. The clinical history of the patient, including lamotrigine exposure and COVID-19 diagnosis, was documented. A 13-cytokine profile assessment was performed in peripheral blood mononuclear cells from the patient and their parents by using quantitative Real Time-Polymerase Chain Reaction (qRT-PCR). A 6-year-old male patient developed lamotrigine-induced TEN with concomitant COVID-19 affecting 90% of the body surface area. Compared with their parents, who were positive for COVID-19, *IL-6*, *IL-4*, and *IL-12* were modulated (downregulated) by TEN. The cytokine profile showed elevated levels of *IL-1α*, *IL-1β*, *IL-5*, *IL-8*, *NF-κβ*, and interferons (*IFN*; α, β, and γ), indicating a robust antiviral response. The immune profile suggested a hyperactivated immune state that contributed to the severity of the patient’s clinical manifestations, leading to death 18 days after hospitalization. Understanding the immune response is important for developing future targeted therapeutic strategies and improving patient outcomes. Further research is needed to explore the interaction between drug-induced SJS/TEN and infections.

## 1. Introduction

Stevens–Johnson syndrome (SJS) and toxic epidermal necrolysis (TEN) are severe but rare blistering disorders that are characterized by generalized epidermal necrosis of the skin and mucous membranes [1,2]. Classification is based on the body surface area (BSA) affected: SJS affects less than 10% of the BSA, while TEN affects more than 30% of the BSA, and an involvement between 10% and 30% is defined as SJS/TEN overlap [3]. SJS/TEN are associated with considerable morbidity and mortality [1]. In Mexico, there are 10.8 cases per million inhabitants of SJS/TEN, with a hospital mortality of 5.8% for the former and 10.4% for the latter [4]. The etiology of SJS/TEN is multifactorial, involving genetic, immunological, and environmental factors [5]. Most TEN cases are drug-induced, with common culprits including antibiotics (sulfonamides, penicillins), anticonvulsants (lamotrigine, carbamazepine), and nonsteroidal anti-inflammatory drugs (NSAIDs) [6]. SJS/TEN manifestations occur between 4 and 28 days after drug exposure [7]. Viral infections, such as herpes simplex and SARS-CoV-2, can also trigger TEN [8,9]. Genetic susceptibility is linked to certain HLA (human leukocyte antigen) alleles, like HLA-B*1502 in Asian populations [10]. The pathogenesis involves a cytotoxic immune response where T cells and natural killer (NK) cells induce keratinocyte apoptosis, leading to widespread epidermal necrosis. This process is mediated by pro-inflammatory cytokines and effector molecules, such as Tumor necrosis factor-alpha (*TNF-α*), interferons, and granzyme B, which contribute to the severity of the disease [11]. The co-occurrence of SJS/TEN and COVID-19 presents a unique challenge due to overlapping inflammatory pathways. The aim of this study was to describe the case of a patient with TEN triggered by lamotrigine and concurrent COVID-19, and to evaluate the cytokine profile for identifying cytokines involved in the pathogenesis of this condition.

## 2. Case Report

**Figure 1:**
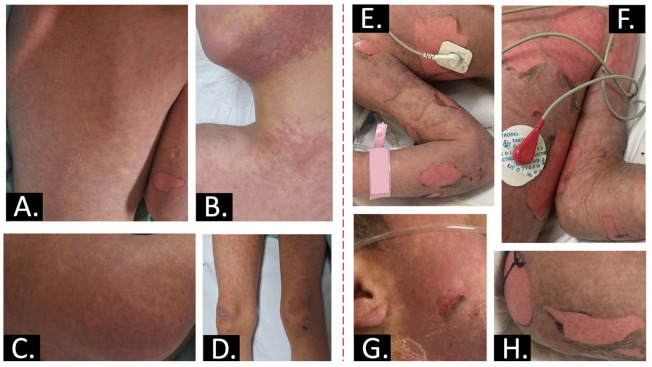
Toxic epidermal necrolysis clinical spectrum. (**A**–**D**): exanthematous rash. Lesions start on the face and thorax before spreading to other areas and are symmetrically distributed. Early lesions typically begin with ill-defined, coalescing, erythematous macules; (**E**–**H**): extensive, sheet-like detachment and erosions, and Nikolsky sign is present.

A 6-year-old male patient with a complete immunization record (excluding COVID-19) and a history of cerebellar syndrome secondary to cortical dysplasia diagnosed at 9 months of age and treated with magnesium valproate, presented with fever, anorexia, and a violaceous erythematous papular rash with a positive Nikolsky sign on the face (sparing the eyes), anterior and posterior thorax, abdomen, buttocks, genitalia, and extremities (sparing the palms and soles). Additionally, there were areas of eroded skin, predominantly on the extremities, as well as blisters, which were most prevalent on the forearms and legs (sparing the palms and plants). The condition affected approximately 87% of the BSA. Symptoms began 14 days after the addition of 25 mg of lamotrigine, administered every 24 h (1.4 mg/kg/day) to the standard treatment regimen of the patient. The patient presented with a skin rash and erythema that was neither pruritic nor painful (Figure 1). Consequently, the decision was made to discontinue the offending drug. Following the onset of the rash and erythema, a period of three days elapsed, the patient developed a fever and exhibited further cutaneous involvement. At this point, treatment with diphenhydramine, ibuprofen, and paracetamol was initiated. The following day, the patient developed blistering lesions on the oral mucosa and extremities, accompanied by significant anorexia for a period of 24 h. This led to the decision to hospitalize the patient.

Upon admission, the patient exhibited a fever of 39.4 °C, tachycardia, and systolic and diastolic blood pressure above the 99th and 95th percentile, respectively. The patient was alert but irritable with no observed neurological deficits. Additionally, the patient showed signs of bilateral conjunctivitis and mucositis, as well as a cough phlegm production, accompanied mild respiratory distress. Laboratory studies revealed the presence of normochromic normocytic anemia, prolonged coagulation times, elevated inflammatory parameters with neutrophilia and lymphocytopenia, hyperglycemia, mild hypokalemia and hypocalcemia, as well as significantly elevated aspartate aminotransferase (AST), lactate dehydrogenase (LDH), creatine kinase (CK), and creatine kinase-MB (CKMB) (Table 1; day 1 and 2).

The patient was transferred to the intensive care unit, where the treatment initiated was similar to that for a major burn patient. A right central venous catheter was placed. Biological samples were obtained for blood bacterial cultures, and a general urine test was indicated. A screening for SARS-CoV-2 (PCR and antibodies) and a cytokine panel were also requested. At this time, a management was started with parenteral fluids at 3000 mL/m^2^ equivalent to 150% of his Holliday–Segar maintenance fluid requirements. The administered fluids contained potassium at a concentration of 30 mEq/L and calcium at a total dose of 1.6 g/day. The management also included ventilatory support via nasal prongs at 3 L/min due to mixed acid–base disorder with hypoxemia, antibiotic with cefalotin (100 mg/kg/day), immunoglobulin at 2 g/kg/dose, and analgesia with buprenorphine (1 μg/kg/h). The bacterial blood and urine cultures were negative. The results of the antibody test against SARS-CoV-2 indicated the presence of IgM antibodies and a titer of 229 U/mL for IgG (reference value < 200 U/mL); however, the quantitative real-time polymerase chain reaction (qRT-PCR) was negative.

**Figure 2:**
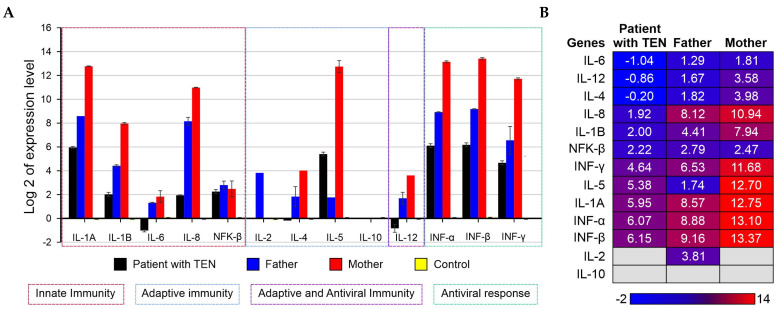
Expression profile of evaluated cytokines. Figure shows the expression level of a 13-citokine panel for the patient with toxic epidermal necrolysis (TEN) and concurrent COVID-19, and for his parents, grouped by immune response in which they participate (**A**); and according with their status of overexpression and underexpression profile (**B**). Expression levels were calculated by quantitative real-time polymerase chain reaction by using GAPDH as endogenous control and RNA of peripheral blood mononuclear cell obtained from healthy controls (with negative qRT-PCR for SARS-CoV-2) as calibrator.

The expression levels of a 13-cytokine panel (*IL-1α*, *IL-1β*, *IL-2*, *IL-4*, *IL-5*, *IL-6*, *IL-8*, *IL-10*, *IL-12*, *NF-κβ*, and *IFN-α*, *IFN-β*, and *IFN-γ*) were measured by qRT-PCR from peripheral blood mononuclear cells (PBMC) obtained from the patient with TEN and their parents (which were positive for COVID-19). The cytokine expression levels were calculated using the 2^−ΔΔCq^ method [12], with glyceraldehyde-3-phosphate dehydrogenase (*GAPDH*) as the endogenous control. RNA sample obtained from PBMC donned by a healthy control (with negative qRT-PCR for SARS-CoV-2) was used as calibrator. The obtained results are displayed in Figure 2A,B. Compared with their parents, *IL-4*, *IL-6*, and *IL-12* were only modulated (downregulated) in the case with TEN, and therefore, it can be considered as modulated specifically by this disease. Considering the control as reference, both in the child and in their parents, the cytokines with elevated levels were *IL-1α*, *IL-1β*, *IL-5*, *IL-8*, *NF-κβ*, and interferons (*IFN*; α, β, and γ), indicating a robust antiviral response initially caused by COVID-19 infection, and probably exacerbated by the lamotrigine-induced TEN in the patient.

During the hospital stay, the patient experienced fever peaks that were difficult to control. Fresh frozen plasma (at a dose of 10 mL/kg) and vitamin K (at 10 mg every 24 h) were administered to normalize clotting times and INR. Parenteral nutrition was started, fluids were adjusted to 2500 mL/m^2^, and control laboratories were requested (Table 1; day 2). He was evaluated by the dermatology service, which prescribed methylprednisolone 1 mg/kg/day and ointments for the skin and mouth containing madecasoside, soothing panthenol, fusidic acid as an antibacterial agent, and Rhealba oatmeal. The ophthalmology service prescribed lubricating drops and cleaning of the eyelids and eyelashes every 4 h with wet gauze for the treatment of viral conjunctivitis. He was evaluated by the cardiology service, which performed an echocardiogram that showed a structurally healthy heart with no hemodynamic abnormalities and a left ventricular ejection fraction (LVEF) of 74%. After 3 days of hospitalization, the patient continued to experience fever peaks up to 39.5 °C along with significant pain. A central blood culture was taken, and he was reevaluated by the infectious disease team, who changed the antibiotic to piperacicline with tazobactam 300 mg IV every 8 h and pain clinic; they also changed the analgesia from buprenorphine to an infusion of 27 mg morphine, 40 mg lidocaine, and 0.5 mg magnesium sulfate, with good response. Given the progression and severity of the mucocutaneous lesions, the liver damage due to elevated transaminases, and clinical and laboratory data indicating a significant inflammatory response (Table 1; day 1 to 4), the patient was transferred to a tertiary care unit.

Upon his admission, the total body surface area affected was recalculated and found to have increased to 90%. The affected area included the posterior thorax, comprising the total back and buttocks in a multiform manner, affecting the dermis and superficial epidermis. The upper extremities and face exhibited second-degree superficial and deep involvement. The lower extremities demonstrated multiform involvement in the epidermis and superficial dermis. The surgical debridement of wounds was conducted, hemostasis was achieved with an adrenaline solution, and a porous cellulose membrane was placed in the four extremities, with Vaseline gauze in the remaining areas. The wounds were then covered with gauze and bandaged. During the procedure, the left central venous catheter was removed and a right one was inserted. The Foley catheter was replaced, and cultures were obtained. These included a central blood culture, and a peripheral blood culture. Additionally, the tip of the removed catheter, and a urine sample showed no evidence of bacterial growth. The central blood culture was positive for *Enterococcus faecium* resistant to beta-lactams, penicillins, cephalosporins, aminoglycosides, and vancomycin, with a minimum inhibitory concentration < 0.5 to erythromycin, ciprofloxacin, tigecycline. A course of management with ceftriaxone (100 mg/kg/day) and ampicillin (150 mg/kg/day) was initiated, and the patient was admitted to the intensive care unit. The patient was managed as a major burn patient with a high fluid intake, enteral support via a nasojejunal tube, analgesia, antibiotic therapy, and general care. He was re-evaluated by the pediatric infectious disease team because the data for the systemic inflammatory response due to tachycardia and fever peaked up to 40 °C, which was difficult to control, so antibiotics were upgraded to meropenem (60 mg/kg/day) and vancomycin (60 mg/kg/day). Two additional debridements were performed as described above (on days 10 and 13 of hospitalization) with significant improvement in and partial epithelialization of the lesions. Following the completion of debridement procedures, the patient exhibited indications of an inflammatory response and shock (fever, tachycardia, and hypotension). Consequently, a new central blood culture was obtained with positive results for *Acinetobacter baumannii*. The antibiotic regimen was modified to include vancomycin (60 mg/kg/day) and colistin (5 mg/kg/day), which resulted in a notable improvement in the patient’s condition over the subsequent days. On the 15th day of hospitalization, the patient exhibited clinical deterioration, manifesting as signs of septic shock, hemodynamic instability, severe thrombocytopenia, and active gastrointestinal bleeding. This necessitated the administration of blood products (erythrocyte concentrates, platelets, and fresh frozen plasma). In the nocturnal period, the patient exhibited asystole, necessitating the administration of two cycles of advanced cardiorespiratory resuscitation maneuvers. Additionally, advanced airway management and vasopressor management with adrenaline and norepinephrine were undertaken. A central blood culture was obtained (*Pseudomonas aeruginosa* positive). The patient’s condition demonstrates evidence of a sluggish evolutionary process, accompanied by the presence of a systemic inflammatory response, hypothermia, acute renal injury, coagulation disorders, and hydroelectrolyte imbalances, including severe hypernatremia, severe hypokalemia, hypocalcemia, and hypochloremia, in addition to hyperlactatemic metabolic acidosis. He ultimately succumbed to septic shock and multiple organ dysfunction syndrome resulting from the bacterial infection. This occurred 16 days after he was admitted to the hospital for first time and 38 days after he was administered lamotrigine.

The immune profile suggested a hyperactivated immune state, which potentially contributed to the severity of clinical manifestations, such as the percentage of body surface area and mucous membranes involved, as well as a persistent fever of difficult control.

## 3. Discussion

SJS/TEN is a rare and potentially fatal disease [2]. The literature reveals a scarcity of cases where a drug has triggered a reaction in conjunction with a concomitant diagnosis of COVID-19 [13,14,15]. Notably, none of these cases were triggered by lamotrigine. In a review conducted between 2019 and 2020, Zou and colleagues identified 22 cases of SJS and TEN secondary to SARS-CoV-2 infection [15]. According to data from the World Health Organization, almost 25 million cases of COVID-19 recorded during this period [16]. This would equate to one case of SJS/TEN for every 1,136,363 individuals.

**Figure 3:**
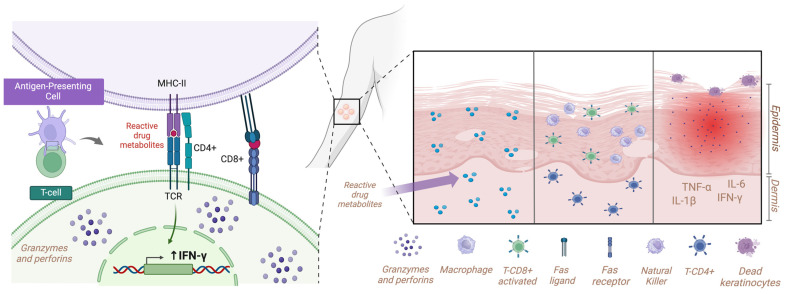
Cell and immune pathophysiology of TEN. Infiltration of the epidermis by activated T lymphocytes (CD8+ epidermis; CD4+ dermis) and natural killer cells induce an immune response against the drug-reactive metabolites. TCRs recognize the molecules and produce interleukins (mainly TNFα) which cause epidermal detachment secondary to keratinocyte apoptosis induced by granzymes, perforins, and Fas/Fas ligand. MHC-II: major histocompatibility complex class II; TCR: T lymphocyte receptor; IFNγ: interferon gamma; TNFα: tumor necrosis factor alpha; IL: interleukin.

The pathogenesis of SJS/TEN is still unclear. However, the infiltration of the epidermis by activated lymphocytes, predominantly CD8+ cells and macrophages, has been observed (Figure 3). It can be hypothesized that an immune reaction against drug-reactive metabolites produced in excess is a potential cause [2]. Given the relatively low number of infiltrating cells present, it is probable that these cells and keratinocytes will not be responsible for the localized cell death, elevated body temperature, and general malaise observed [17].

A number of non-mutually exclusive models have been put forth to elucidate the manner in which small molecular synthetic compounds are detected by T cells in an MHC-dependent manner [2]. These include the hapten concept/prohapten model, the p-i model, and the altered repertoire model [18]. Anemia and lymphopenia were common clinical findings in a series of cases of TEN, whereas eosinophilia was less frequently observed [19]. The presence of neutropenia was indicative of a poor prognosis, whereas the recovery of the granulocyte count to normal levels suggests a more favorable outcome [19]. Nevertheless, the underlying cause of granulocytopenia remained undetermined [20], consistently with the overcome of our patient.

Regarding the clinical management of patients with TEN, Varol and colleagues presented a case of two pediatric patients, both aged six years old, who developed TEN as a consequence of contracting SARS-CoV-2 [9]. The first child exhibited 32.5% body surface area involvement (face, chest, abdomen, genitals, upper limbs) and a co-infection with Influenza A. The second child demonstrated 44.5% involvement (face, trunk, distal extremities, oral mucosa) and was using Augmentin. Both patients underwent plasma exchange therapy. Despite this treatment and subsequent recovery, the first child was discharged after 12 days of hospitalization, whereas the second child required an additional 9 days for discharge [9]. Similarly, in the case of a 6-year-old boy who presented with NET secondary to ibuprofen and a concomitant SARS-CoV-2 infection, the patient was treated with intravenous immunoglobulin, dexamethasone, oral prednisolone, and cyclosporine, showing a favorable evolution within one week, with recovery of the skin and mucous membranes observed at one month [19]. These findings suggest that a treatment strategy that aim to “aggressively” mediate the inflammatory response (even when it is exacerbated) may yield more favorable outcomes than those that do not in cases of significant body surface area damage. In a study of drug-induced immune reactions, the levels of *TNF-α*, granzyme B, and perforin during the acute phase of the reaction in SJS/NET were higher than normal, while these levels returned to normal after the reaction had resolved [11]. The fundamental elements of the therapeutic regimen of this kind of patients are wound care and supportive care. The literature indicates that the use of intravenous immunoglobulins and steroids, cyclosporine, plasmapheresis, and *TNF-α* inhibitors has been associated with a reduction in mortality and morbidity in pediatric patients [21]. In a study conducted in Ankara, the dermatological manifestations associated with SARS-CoV-2 infection in pediatric patients were evaluated. Of the 5143 infected children, only 13 (0.25%) developed skin lesions, with maculopapular exanthema (61.5%) and urticaria (23%) being the most frequent. Among the cohort, only one child developed SJS secondary to amoxicillin–clavulanate administration and subsequently died [13]. In our study, we found that the interleukins primarily involved in TEN were *IL-4*, *IL-6*, and *IL-12*, which were modulated (downregulated) by TEN, along with augmented *IL-1α*, *IL-1β*, *IL-5*, *IL-8*, *NF-κβ*, and interferons (*IFN*; α, β, and γ), which were modulated by COVID-19 (Table 2).

If the determination of *TNF-α* was not available in our study, *TNF-α* activates the *NF-κβ* pathway (as seen in our study), which is important for the transcription of various pro-inflammatory genes, including those encoding *TNF-α* itself, namely, *IL-1β*, *IL-6*, and *IL-8*. This creates a feedback loop that perpetuates and amplifies the inflammatory response. *IL-4* and *IL-10* are anti-inflammatory cytokines that can modulate the effects of *TNF-α* by promoting a Th2 immune response and inhibiting the production of pro-inflammatory cytokines. However, in the context of SJS/TEN/COVID-19, and in agreement with our study, their levels are often insufficient to counterbalance the overwhelming pro-inflammatory response. In the same way, despite *NF-κβ* upregulation, the low levels of *IL-6* observed in this study may be explained by post-transcriptional regulation, which could limit *IL-6* expression [22]. Additionally, competition between pro-inflammatory pathways or the influence of anti-inflammatory cytokines, such as *IL-10* and *TGF-β*, may contribute to this downregulation [22,23] (Table 2).

Regarding the use of *TNF-α* inhibitors, in a previous study, Zhang et al. examined 21 case reports, 4 case series, and 2 randomized controlled trials (RCTs) analyzing *TNF-α* inhibitor use, reporting positive results in 86.8% of patients [24]. *TNF-α* inhibitors, such as Infliximab, could theoretically have been beneficial in our case due to their ability to reduce inflammation and apoptosis mediated by *TNF-α* [2]. This could help in decreasing the severity of the skin and mucosal lesions. However, the potential risks, particularly in the context of concurrent COVID-19 infection, would need to be carefully weighed. This is because *IL-1α*, *IL-1β*, *IL-5*, *IL-8*, *NF-κβ*, and interferons (α, β, and γ), are molecules that overlap with the immune response to COVID-19. Further research and clinical trials are needed to establish the safety and efficacy of *TNF-α* inhibitors in the treatment of SJS/TEN, particularly in patients with coexisting infections.

### Study Limitations and Perspectives

When interpreting our findings, potential confounding factors such as age-related immune variability, differences in lymphocyte subpopulations within PBMCs, and secondary infections due to the compromised skin barrier should be considered. Although cytokine expression levels were normalized using a healthy control (a young adult with a negative COVID-19 PCR), these limitations remain. The low incidence of SJS/TEN in Mexico, estimated at 10.8 cases per million inhabitants, underscores the rarity of this condition and the challenge of assembling a comparative cohort. This being the first recorded case in our hospital further limits the feasibility of a case–control study.

As a case report, our objective was to describe the clinical and molecular characteristics rather than establish causality. Future multicenter studies may help explore the impact of COVID-19 on SJS/TEN pathogenesis. Despite the potential value of serum *IL-6* measurements, our study prioritized mRNA expression in PBMCs as a practical and informative approach within clinical constraints. These findings highlight the uniqueness of the case and the need for further research on the interplay between drug-induced SJS/TEN and viral infections.

## 4. Conclusions

In conclusion, coinfection with SARS-CoV-2 in patients with TEN, though uncommon, poses a significant risk by intensifying the inflammatory response to potentially life-threatening levels. This study revealed that *IL-4*, *IL-6*, and *IL-12* play key roles in drug-induced TEN, while COVID-19 infection may lead to elevated levels of *IL-1α*, *IL-1β*, *IL-5*, *IL-8*, *NF-κβ*, and interferons (*IFN*; α, β, and γ). Consequently, treatments that focus on inducing tolerance molecules and/or the inhibition of over-expressed inflammatory molecules could reduce lesion severity and duration, ultimately improving patient outcomes and lowering the risk of complications.

## Figures and Tables

**Table 1:** Laboratory findings observed during the hospitalization of the patient.

Parameter	Reference	Hospitalization Days			
		Day 1	Day 2	Day 3	Day 4
Red blood cells					
Erythrocyte (10^6^/μL)	3.0–5.3	3.87	3.61	NT	3.62
Hemoglobin (g/dL)	12.6–16.0	11.6 *	11.1 *	NT	11.1 *
Hematocrit (%)	42.0–51.0	35.3 *	33.1 *	NT	33.9 *
Mean corpuscular volume (fL)	80.0–100.0	91.2	91.7	NT	93.6
MCH (pg/cell)	27.0–32.0	30	30.7	NT	30.7
MCHC (g/dL)	32.0–36.0	32.9	33.5	NT	32.7
Red cell distribution width (%)	11.0–17.0	14.1	14.3	NT	14
Platelets (10^3^/μL)	150.0–400.0	150	119 *	NT	162
Mean platelet volume (fL)	7.0–11.0	9.1	9.6	NT	8.3
White blood cells					
Leukocytes (10^3^/μL)	5.0–10.0	6.38	3.9 *	NT	3.6 *
Neutrophils (%)	37.0–75	82.5 *	74.2	NT	50.7
Lymphocytes (%)	17.0–45.0	10.7 *	16.4 *	NT	35.7
Monocyte (%)	2.0–12.0	6.4	9.4	NT	13.6 *
Eosinophils (%)	1.0–7.0	0.2 *	NT	NT	NT
Basophils (%)	0.3–2.0	0.2 *	NT	NT	NT
Leukocyte absolute values					
Lymphocytes (10^3^/μL)	1.0–5.0	0.68 *	0.6 *	NT	1.3
Neutrophils (10^3^/μL)	2.0–8.0	5.27	2.9	NT	1.8 *
Monocyte (10^3^/μL)	0.1–1.0	0.41	0.4	NT	0.5
Eosinophils (10^3^/μL)	0.0–0.4	0	NT	NT	NT
Basophils (10^3^/μL)	0.0–0.2	0	NT	NT	NT
Leukocyte differential					
Lymphocytes (%)	NA	17 *	NT	NT	NT
Monocyte (%)	NA	9	NT	NT	NT
Segmented neutrophils (%)	NA	40 *	NT	NT	NT
Bands (%)	NA	34 *	NT	NT	NT
Eosinophils (%)	NA	0	NT	NT	NT
Basophils (%)	NA	0	NT	NT	NT
Coagulation parameters					
Prothrombin time (seconds)	11.0–14.0	22.9 *	NT	15.7 *	NT
International normalized ratio (%)	0.93–1.5	1.77 *	NT	1.18	NT
PTT (seconds)	26.0–40.0	40.7 *	NT	35.4	NT
D dimer (μg/mL)	<0.50	1.37	NT	NT	NT
Fibrinogen (mg/dL)	200–400	274	NT	NT	NT
Biochemical parameters					
Ferritin (ng/mL)	30–400	397.6	NT	NT	NT
Glucose (mg/dL)	70.0–110.0	147 *	112 *	126 *	121 *
Urea (mg/dL)	15.0–43.0	18.7	18.3	17.7	20.8
Serum creatinine (mg/dL)	0.7–1.5	0.36	0.27 *	0.25 *	0.28 *
Blood urea nitrogen (mg/dL)	7.0–20	9	8.6	NT	10
Sodium (mmol/L)	135.0–148.0	140	138.3	137	146
Potassium (mmol/L)	3.5–5.3	3.04 *	3.9	3.57	4.15
Chlorine (mmol/L)	98.0–107.0	106.5	108.7 *	106.5	110.4 *
Calcium (mg/dL)	8.4–10.2	7.15	9.2	7.27 *	7.81 *
Phosphorous (mg/dL)	2.5–4.5	3.2	3.3	2.99	3.48
Magnesium (mg/dL)	1.6–2.3	1.7	1.6	1.74	1.98
Bilirubin total (mg/dL)	0.2–1.3	0.19	0.19	NT	0.22
Direct bilirubin (mg/dL)	0.0–0.4	0.1	0.09	NT	0.13
Indirect bilirubin (mg/dL)	0.0–1.0	0.09	0.1	NT	0.09
AST (U/L)	10.0–42.0	55 *	67.4 *	NT	63.4 *
ALP (U/L)	38.0–126.0	98	83.7	NT	95
ALT (U/L)	0.0–42.0	18.9	27.1	NT	35.2
LDH (U/L)	135.0–225.0	712 *	512 *	NT	451 *
Globulin (mg/dL)	2–3.3	1.96 *	2.99	NT	2.5
Albumin (g/dL)	3.8–5.10	3.02 *	2.99 *	2.65 *	2.94 *
Albumin globulin ratio	1.10–18.10	1.54	0.94 *	NT	1.18
Total protein (g/dL)	6.4–8.3	4.98 *	5.8 *	NT	5.44 *
CKMB (U/L)	7.0–25.0	437.2 *	NT	NT	NT
CK (U/L)	20.0–180.0	311 *	NT	NT	NT
Triglycerides (mg/dL)	40.0–160.0	NT	78	NT	86
Total cholesterol (mg/dL)	50.0–200.0	NT	91	NT	121
HDL cholesterol (mg/dL)	40.0–45.0	NT	38.5 *	NT	46.1
LDL cholesterol (mg/dL)	50.0–172.0	NT	75.4	NT	94.6
VLDL cholesterol (mg/dL)	45.0–65.0	NT	15.6 *	NT	17.2 *
C reactive protein (mg/dL)	0.25–0.65	NT	NT	NT	11.98 *
Procalcitonine (ng/mL)	<0.5	NT	NT	NT	0.781

MCH: mean corpuscular hemoglobin; MCHC: mean corpuscular hemoglobin concentration; NA: not applicable; PTT: partial thromboplastin time; AST: aspartate aminotransferase; ALP: alkaline phosphatase; ALT: alanine aminotransferase; LDH: lactate dehydrogenase; CKMB: creatine kinase MB; CK: total creatine kinase; NT: no tested. Altered values are indicated with an asterisk.

**Table 2:** Comparative analysis between expected and observed cytokine expression levels in a patient with toxic epidermal necrolysis and COVID-19.

Cytokine	Type of Immune Response	Types of Producing Cells	Expected Level	Observed Level	Possible Causes of/Possible Explanations for Discrepancy
IL-1a	Innate	Monocytes, macrophages	↑	↑	Severe inflammation from TEN
IL-1b	Innate	Monocytes, macrophages	↑	↑	Severe inflammation from TEN
IL-6	Innate	Monocytes, macrophages, T cells	↑	↓	Acute inflammation from TEN and COVID-19/individual variability, disease phase, viral effect
IL-8	Innate	Monocytes, macrophages, T cells	↑	↑	Inflammation and neutrophil attraction
NFkβ	Innate	All cells	↑	↑	Pro-inflammatory genes regulation
IL-2	Adaptive	T cells	↑	ND	T cell activation/Immunosuppression or individual variability
IL-4	Adaptive	Th2, mast cells	Variable	↓	Th2 response/immunosuppression, individual variability
IL-5	Adaptive	Th2, eosinophils	Variable	↑	Th2 response and eosinophils/inflammation and Th2 response
IL-10	Adaptive	Monocytes, macrophages, Th2	↑	ND	Anti-inflammatory regulation/insufficient regulatory response
IL-12	Adaptive, Antiviral	Dendritic cells, macrophages	↑	↓	Antiviral and Th1 response/viral evasion, individual variability
INFα	Antiviral	Dendritic cells, macrophages	↑	↑	Antiviral response
INFβ	Antiviral	Dendritic cells, fibroblasts	↑	↑	Antiviral response
INFγ	Antiviral, Adaptive	T cells, NK cells	↑	↑	Antiviral response and macrophage activation

ND: none detected; TEN: toxic epidermal necrolysis; Th2: T helper cells. ↑: increased level, ↓: decreased level.

## Data Availability

The original contributions presented in this study are included in the article. Further inquiries can be directed to the corresponding authors.
